# Reduction of cervical cancer incidence within a primary HPV screening pilot project (WOLPHSCREEN) in Wolfsburg, Germany

**DOI:** 10.1038/s41416-019-0453-2

**Published:** 2019-04-16

**Authors:** Johannes Horn, Agnieszka Denecke, Alexander Luyten, Beate Rothe, Axel Reinecke-Lüthge, Rafael Mikolajczyk, Karl Ulrich Petry

**Affiliations:** 1 Institute for Medical Epidemiology, Biometrics, and Informatics, Martin-Luther-University Halle-Wittenberg, Halle, Germany; 2 Department of Obstetrics and Gynecology, Klinikum Wolfsburg, Wolfsburg, Germany; 3 Abts+partner, Frauenärzte, Prüner Gang, Kiel, Germany; 4 Central Laboratory, Klinikum Wolfsburg, Wolfsburg, Germany; 5 Institute of Pathology, Klinikum Wolfsburg, Wolfsburg, Germany; 6 German Centre for Infection Research, Site Braunschweig-Hannover, Germany

**Keywords:** Cancer screening, Cervical cancer, Human papilloma virus

## Abstract

**Background:**

Randomised controlled trials showed human papillomavirus (HPV)-based screening leads to a significant reduction in cervical cancer incidence compared with cytology-based screening only.

**Methods:**

Non-hysterectomised participants ≥30 years underwent co-testing with Papanicolaou (Pap) smear and HR-HPV testing (Hybrid Capture 2; HC2). Women with normal findings had their next screening round after 5 years, and HC2+ and Pap abnormal cases were immediately referred for colposcopy, while cases with discordant findings had repeat testing after 12 months with referral to colposcopy in cases with persistent positive findings.

**Results:**

Twenty-six thousand six hundred and twenty-four women were recruited between February 2006 and December 2016. Two hundred and seventy-four CIN3+ cases were diagnosed (270 HPV+, 4 HPV−), including 31 invasive cervical cancers (29 HPV+, 2 HPV−). No CIN3+ was detected in HPV− women with abnormal cytology. We observed a significant decline in the 5-year incidence of CIN3+ (from 0.96% [95% CI 0.85–1.09%] to 0.16% [95% CI 0.10–0.25%]; *p* < 0.0001) and cervical cancer (from 0.10% [95% CI 0.07%–0.15%] to 0.025% [95% CI 0.01–0.08%]; *p* = 0.01) between the first and subsequent rounds. Approximately 90% (246/274) of CIN3+ cases were diagnosed at first colposcopy.

**Conclusions:**

The decline in disease rates with 5-yearly co-testing seems mainly attributable to HPV testing since no CIN3+ occurred in HPV−/Pap+ women.

## Background

Screening programmes based on the Papanicolaou (Pap) smear have achieved large reductions in cervical cancer incidence and mortality,^1^ particularly in countries with high target population coverage.^2^ However, one of the limitations of cytology-based screening is a low sensitivity for detecting precursor lesions of cervical cancer (cervical intraepithelial neoplasia grade ≥2 (CIN2+))^3,4^; in addition to the need for repeated screening at short intervals, there is a high chance that false-negative findings will be repeated.^5^

Screening for human papillomavirus (HPV) as the causative agent of cervical cancer^6^ was first proposed in the 1990s, prior to the vaccination era, to improve the prevention of this lethal malignancy.^7^ Subsequently, randomised controlled trials showed that HPV testing not only detects more precursor lesions at first screening, but, when coupled with therapy, also leads to a reduction in the incidence of these lesions in the following years and a substantial (70%) reduction in subsequent cases of invasive cervical cancer.^8,9^

An important consequence of the higher sensitivity of HPV testing for CIN2+^10^ is the longer duration of a low-risk period after a negative result, both for high-grade CIN and invasive cancer, enabling safe extension of the intervals between screening episodes.^11–14^ Although randomised trials demonstrated the superiority of HPV-based screening for women aged ≥30–35 years,^9^ there is still little evidence that similar reductions in cervical cancer incidence can be achieved in a real-world setting in unselected women participating in routine screening programmes. Findings from controlled clinical trials may differ from observations in the real-world for several reasons related to selection of subjects and participating healthcare professionals, differences between study protocols and everyday clinical pathways and the lack of a control arm for comparison and mitigation of bias. Here we report 11-year follow-up data from a local screening programme based on HPV and cytology co-testing in the Wolfsburg region of Germany. The main focus of this analysis is to determine the incidence of cervical cancer diagnosed in the first screening round compared with subsequent rounds.

## Methods

The **Wol**fsburg pilot project for better prevention of cervical cancer with **p**rimary **H**PV **screen**ing (WOLPHSCREEN) has been described in detail previously.^15^ In brief, WOLPHSCREEN was a locally organised HPV screening programme for non-hysterectomised women aged 30–70 years, covered by ‘Deutsche BKK’ and ‘Audi BKK’ health insurance companies. The upper age limit of 70 years, recommended at the start of the WOLPHSCREEN programme, became mandatory from 2012. Every woman coming for screening could freely choose to do either yearly Pap-smear or Pap-smear and HPV-test with a 5-year interval. Although both possible screening modalities were reimbursed by the health insurance companies, women opted almost entirely for co-testing using Pap smear and HPV (Hybrid Capture 2 (HC2)) tests. The included population was, therefore, unselected and non-randomised. Data were collected in an observational fashion, with all patients co-tested with HC2 and cytology (no control arm) and followed according to regular care using a rigorous, individualised, risk-based follow-up.

The same testing protocols were used for all screening rounds. Women with positive HC2 test results and abnormal Pap smears were referred immediately for colposcopy; those with positive HC2 tests and normal cytology were retested annually and referred for colposcopy if HPV infection persisted, while those with negative HC2 tests and normal Pap smears returned for their next screening round 5 years later. Opportunistic testing did not play a role in the WOLPHSCREEN protocol.^15^

Screening round 1 included all participants recruited between 1 February 2006 and 31 December 2016. Round 1 started with the first primary screening and ended with the second screening round, loss to follow-up or the end of 2017. Screening rounds 2 and 3 covered procedures among eligible women who had completed a planned 5-year screening interval after rounds 1 and 2, respectively. Follow-up data were included up to 31 December 2017. As there was no formal notification that participants had left WOLPHSCREEN or the Wolfsburg area, follow-up data for participants who left the programme because they changed their health insurance provider, reached the study exit age of 70 years, underwent hysterectomy, were diagnosed with other gynaecologic malignancies or left the area were obtained if possible from the tumour registry of Lower Saxony, insurance databases or our internal hospital database to identify cases of invasive cervical cancer occurring outside of WOLPHSCREEN. We also compared all cervical cancer cases registered within the Cancer Centre Wolfsburg 2006–2017 with cases registered by the cancer registry of Lower Saxony for the Wolfsburg region. For the very few cases that were not treated at our institution, we contacted the health insurer, responsible gynaecologist and/or the treating institution to clarify if these cases occurred in former or active WOLPHSCREEN participants. The aim was to identify incident cancer cases occurring up to 6 years after screening to mitigate the risks of underestimation (due to missing diagnosed cancer cases or loss to follow-up) or overestimation (due to inclusion of cancer cases diagnosed after the intended 5-year interval and outside Wolfsburg). To ensure a follow-up period of at least 1 year for each woman included in a screening round, we did not include screening performed after 1 January 2017.

Testing procedures: HC2 tests, done according to the manufacturer’s protocol (Qiagen, Hilden, Germany), detect high-risk HPV subtypes (16, 18, 31, 33, 35, 39, 45, 51, 52, 56, 58, 59 and 68). Abnormal smear test results were classified as Pap IIw or IIp or worse, using the Munich Cytological Classification,^16^ which are equivalent to, or worse than, atypical cells of undetermined significance (ASC-US) in the Bethesda system. After February 2011, optional repeat Pap smear after 6 months for women with initial positive result was replaced with p16/Ki-67 dual-stained cytology test (CINTEC plus, Roche Diagnostics, Germany), as described previously.^17,18^

### Endpoints and statistical analysis

The main objective of this analysis was to determine the rates of invasive cervical cancer and CIN3 or cancer (CIN3+) during the first and subsequent screening rounds. The predefined study endpoints for this analysis were:

- Rates of invasive cervical cancer, CIN3+ and CIN2+ detected in screening round 1 and subsequent screening rounds among the overall population.
- Proportion of participants in defined risk groups (HC2+/Pap abnormal; HC2+/Pap normal and HC2−/Pap abnormal) according to test findings at screening round 1 and subsequent outcomes (invasive cervical cancer, CIN3+ and CIN2+) in these defined risk groups.
- The time between primary screening and diagnosis of CIN2+, CIN3+ or cervical cancer.

Outcomes data for cases of invasive cervical cancer, CIN3+ and CIN2+ over time were reported descriptively. Cases occurring >6 years after the last screening round were excluded from the analysis. In women with multiple outcomes in the same round (e.g. CIN2 and CIN3), the highest (most severe) clinical finding was always used for the analysis. The time to diagnosis was counted as the time to the first diagnosis of any clinical outcome (invasive cervical cancer, CIN3 or CIN2). Confidence intervals (CIs) were calculated using R (Clopper Pearson formula for proportion).

## Results

**Fig. 1:**
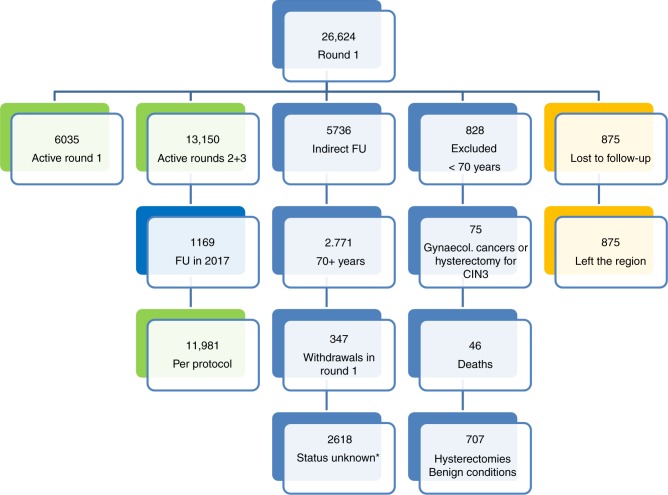
Flow of subjects recruited for screening round 1 and beyond. Reasons for omission are shown by orange highlight. *Status was unknown in these women because, following a merger between Deutsche BKK and BARMER, the combined data set was incomplete, and therefore it was not possible to determine whether these participants left the project or are late attenders

A total of 26,624 women, recruited between 1 February 2006 and 31 December 2016, had primary screening tests in round 1. Figure 1 shows the flow of subjects included in the analysis and reasons for exclusion or omission. At the cut-off date of 31 December 2016, 6035 participants recruited from 1 January 2012 were still in screening round 1 per protocol. Among 20,589 participants recruited to round 1 up to 31 December 2011, entry to round 2 was delayed until 2017 in 1169, and these women were not included in the analysis; however, no case of cervical cancer was observed in this subgroup. A total of 2771 participants were excluded from the analyses of screening rounds 2 or 3 because they had reached the defined maximum age of 70 years, although they continued to be monitored for CIN3+ using available registry data.

We registered 46 deaths not related to cervical cancer in WOLPHSCREEN participants aged <70 years. Overall, 62 participants were treated for endometrial (*n* = 48) or ovarian cancer (*n* = 14), 32 of whom were aged <70 years at the time of diagnosis. All 28 participants diagnosed with cervical cancer in round 1 were excluded from subsequent screening rounds, as were 15 women who had a hysterectomy because of CIN3 or adenocarcinoma in situ of the cervix. Overall, 707 participants aged <70 years with normal cytology and a negative HPV test underwent hysterectomy for benign indications; no cases of CIN2+ were diagnosed on the final histological cervical specimen in this cohort. There were 347 withdrawals from WOLPHSCREEN in screening round 1 and none afterwards. The status of a further 2618 women is uncertain. Because of a merger between Deutsche BKK and BARMER health insurance, we were unable to identify whether these women joined Audi BKK or other health insurance providers, left the project for other reasons or are delayed in attending the next screening round.

The analysis therefore includes per-protocol data from 26,624 women in round 1 and 11,981 in subsequent screening rounds. The median follow-up times were 1644 days in round 1 and 1401 days in subsequent rounds (cumulative).

### Incidence of cervical cancer, CIN3+ and CIN2+

Overall, 274 CIN3+ cases were diagnosed, including 31 invasive cervical cancers among women followed per protocol. Among 28 cases of cervical cancer diagnosed in round 1, 14 were detected within 6 months and 23 within 2 years of recruitment, while 2 patients had a delayed diagnosis because of lack of compliance with colposcopy and/or proposed treatment of CIN3 and 1 patient had a delayed diagnosis because of colposcopy failure. In round 2, three cases of cervical cancer were diagnosed, one within 2 years of entry to round 2. One patient who had HPV persistency after assessment in round 1 had a severely delayed diagnosis in round 2 because of colposcopy failure.

Follow-up of women excluded from WOLPHSCREEN in 2012 or later, using searches of the hospital registry, the cancer registry of Lower Saxony and the health insurance providers’ databases, showed no further cases of invasive cervical cancer among women who reached the age of 70+ years or those who had hysterectomy for benign indications. We identified three cancer cases who were excluded because these occurred >6 years after recruitment to round 1 and never attended for round 2. One of these excluded cases of cervical cancer was diagnosed after 10 years at another institution as a stage IV adenocarcinoma of the cervix and could be a misclassification because there was no histology review or surgical staging performed; misclassifying cancers of other origin as cervical cancer is common without surgical staging.^19^ The two other cases were microinvasive cancers; the first was diagnosed at our institution after 8 years, while another occurred in a patient who underwent CIN3 treatment within WOLPHSCREEN, left the Wolfsburg region and did not attend follow-up examinations as advised and was finally diagnosed with microinvasive cervical cancer.

**Fig. 2:**
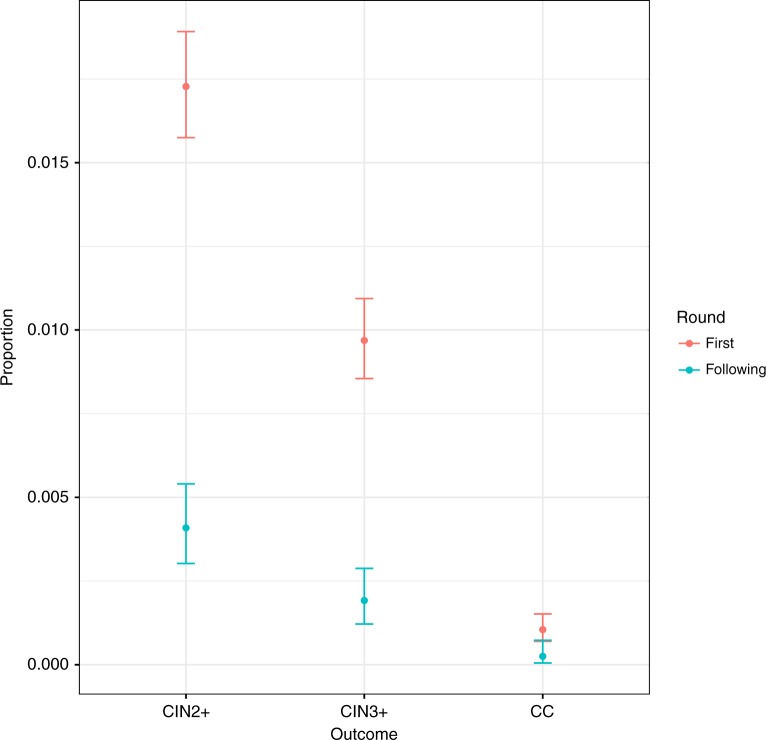
Proportion of women with CIN2+, CIN3+ or cervical cancer (CC) in the first and subsequent screening rounds. (Points show proportion of participants and whiskers the 95% confidence intervals)

Figure 2 shows the rates of cervical cancer, CIN3+ and CIN2+ in round 1 compared with subsequent screening rounds. The incidence of cervical cancer decreased from 0.10% (95% CI: 0.07–0.15%; *n* = 28 cases) in round 1 to 0.025% (95% CI: 0.01–0.08%; n = 3) in subsequent rounds. The difference in cervical cancer incidence between the first and the subsequent screening rounds was significant (Fisher exact test *p* = 0.01). The incidence of CIN3+ was 0.96% (95% CI: 0.85–1.09%; *n* = 255/26,624) in round 1 and dropped to 0.16% (95% CI: 0.10–0.25%; *n* = 19/11,947) in subsequent screening rounds (*p* < 0.0001).

As the CIN3 incidence declines with age in a population aged 30–70 years, we did an additional sensitivity analysis excluding participants aged 30−34 years, which confirmed declines between round 1 and subsequent rounds for cervical cancer (0.11 in round 1 and 0.03 in subsequent rounds, *p* = 0.007), CIN3+ (0.69 and 0.19, *p* < 0.0001) and CIN2+ (1.30 and 0.43, *p* < 0.0001).

### Proportion of participants in defined risk groups and associated outcomes

The HC2+/Pap-abnormal group comprised 284 women in round 1 and 37 in subsequent rounds, the HC2+/Pap-normal group comprised 1699 and 511 women, respectively, the HC2−/Pap-abnormal group 309 and 119 women, respectively, and the HC2−/Pap-normal group 24,362 and 11,356 women, respectively. Between round 1 and subsequent rounds, there was a 5-fold decease in the proportion of women in the HC2+/Pap-abnormal group and a 1.5-fold decrease in the HC2+/Pap-normal group.

The proportion of women diagnosed with cervical cancer decreased between round 1 and subsequent rounds from 5.28% (15/284) to 0% (0/37) in the HC2+/Pap-abnormal group and from 0.71% (12/1699) to 0.19% (1/518) in the HC2+/Pap-normal group. No cases of CIN3+ or cervical cancer were diagnosed in the HC2−/Pap-abnormal group (0/309 in round 1 and 0/119 in subsequent rounds). In the HC2−/Pap-normal group, 1 case was diagnosed among 24,362 women within round 1 and another 1 among 11,356 women during subsequent screening rounds (incidence of 0.041/1000 and 0.088/1000, respectively).

Overall, 270 of the 274 CIN3+ cases were HC2+ and 4 were HC2−, while 29 of the 31 invasive cervical cancers were HC2+ and 2 were HC2−.

### Time between primary screening and diagnosis

**Fig. 3:**
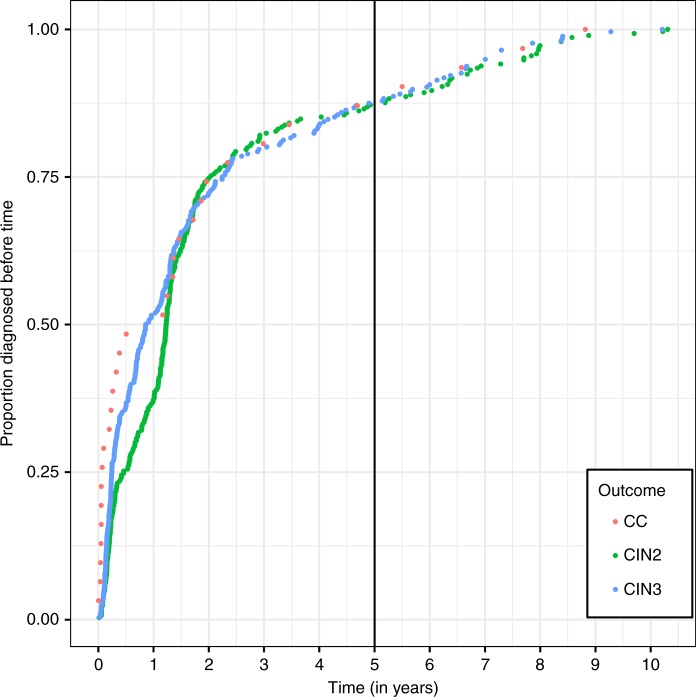
Time from round 1 to the diagnosis of CIN2, CIN3 or cervical cancer. Each dot represents the diagnosis of an individual participant

Figure 3 shows the time from primary screening in round 1 to the diagnosis of cervical cancer, CIN3+ and CIN2+. Approximately 90% of cervical cancer (28/31) and CIN3+ (246/274) cases were diagnosed at first colposcopy. The incidence of outcomes in round 1 was defined as the incidence within 5.5 years of recruitment to WOLPHSCREEN; however, six cases of CIN2+ (no cancers) detected sooner than 5.5 years after their primary screening test were diagnosed in the second follow-up round because the women had already had their second primary screening round at 5.0–5.4 years.

**Fig. 4:**
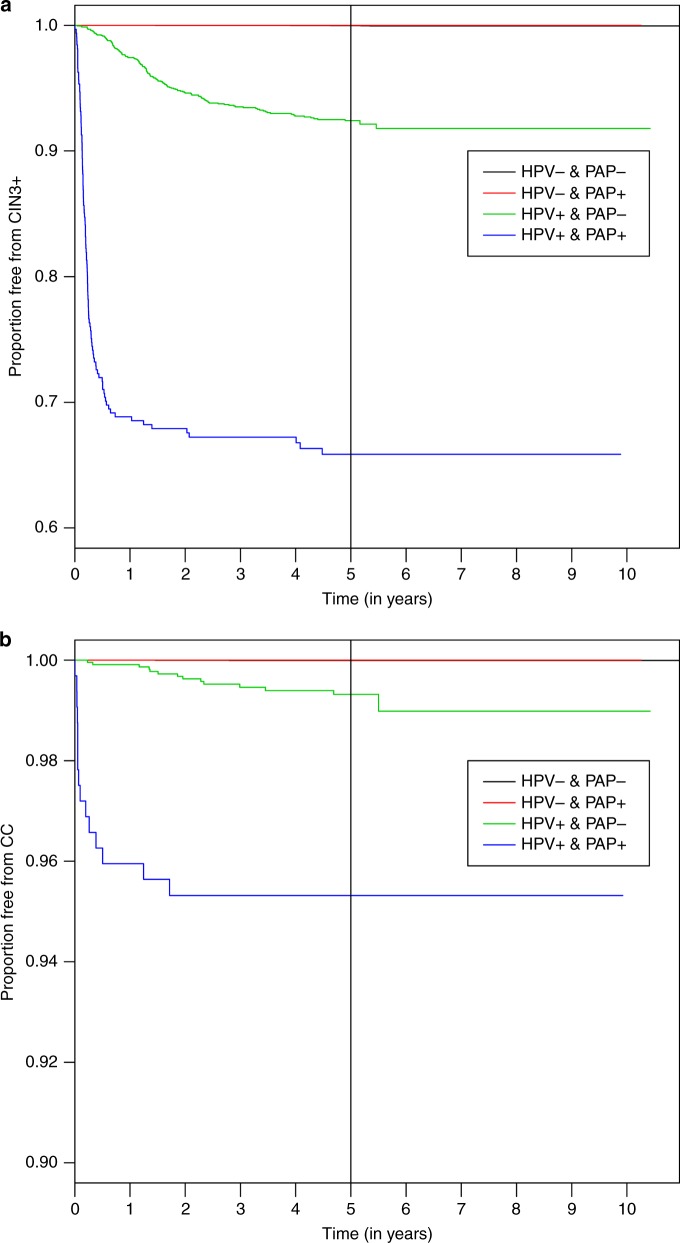
Kaplan–Meier curves showing the proportion of patients developing **a** CIN3+ and **b** cervical cancer, according to prespecified risk group based on human papillomavirus and Papanicolaou status

Figure 4 shows the cumulative incidence of CIN3+ over time according to HPV and Pap test results. CIN3+ occurred almost exclusively in HPV-positive women and most (90%) were detected within round 1 screening. During long-term follow up, the risks of CIN3 (Fig. 4a) and cervical cancer (Fig. 4b) were significantly lower among HPV-negative women than among those with positive HPV tests, irrespective of Pap cytology (Fisher test *p* < 0.0001) and no cases of CIN3 were detected in the HC2−/Pap-abnormal group.

## Discussion

This locally organised HPV-based screening programme with individualised, risk-based follow-up protocol was implemented to improve detection of cervical cancer in a real-world setting. The current analysis with 11 years follow-up shows that co-testing detects approximately 90% of precursor lesions and invasive cervical cancer at first screening leading to a decline in disease rates in subsequent screening rounds.

Randomised clinical trials previously demonstrated improved outcomes with HPV screening compared with Pap cytology.^8,9,20^ The ‘HPV FOCAL Randomised Clinical Trial’ including 19,009 women compared the cumulative incidence of CIN3+ using primary HPV testing alone or liquid-based cytology (both groups had HPV and liquid-based cytology co-testing at the 48-month exit point). The incidence of CIN3+ at 48 months was lower in the HPV testing group (2.3/1000 [95% CI, 1.5–3.5] versus 5.5/1000 [95% CI, 4.2–7.2], respectively).^20^ An analysis of another four randomised trials (Swedescreen, POBASCAM, ARTISTIC and NTCC) comprising a total of 176,464 women aged 20–64 years followed for a median of 6.5 years showed that HPV screening significantly reduced the incidence of invasive cervical cancer beyond 2.5 years compared with cytology-based screening.^9^ At 5.5 years, the cumulative incidence of invasive cervical carcinoma (per 100,000) in women with negative tests at entry was 8.7 with HPV screening compared with 36.0 with cytology. In the WOLPHSCREEN analysis, the cumulative incidence of invasive cervical cancer was 4.1 per 100,000 in women with a HC2− test and normal Pap cytology at round 1, which was even lower than the estimate for the HPV test only from the analysis of randomised trials. Although there is an inherent risk of bias in single-arm, observational studies in unselected populations, it is reassuring that WOLPHSCREEN provided evidence to support an HPV-based screening approach. Since the WOLPHSCREEN project started, Germany has decided to switch from yearly Pap screening to Pap and HPV co-testing with a 3-year interval for 35–60-year-old women in 2020. The 3-year interval was a political decision. Data from randomised controlled trials show that the screening interval can be extended up to 5 years,^9^ and this recommendation is supported by the WOLPHSCREEN data.

There was a notable reduction in the incidence of precursor lesions between round 1 and subsequent screening rounds, particularly for women with an initially positive HPV test. The reduction in the incidence of cervical cancer in subsequent rounds can be explained by improved detection of precursor lesions during round 1 screening, allowing at-risk individuals to be referred for colposcopy and definitive treatment where indicated. Of note, 28 cases of cervical cancer were associated with HPV infection present at the time of recruitment. Moreover, other HPV tests showed that two HC2-negative cases tested positive for HPV-DNA in the tumour tissue and the initial sample. Therefore, only 1 case, a microinvasive adenocarcinoma diagnosed 7.5 years after recruitment, may have developed from a newly acquired HPV infection. Long-term follow-up data showed a very low risk of CIN3+ among women with negative HPV tests. During the 11-year follow-up, we did not find any cases of CIN3 or invasive cervical cancer in women with a negative HPV test and abnormal cytology. Our data suggest that the risk of CIN3+ and cervical cancer is determined mainly by HPV status, whereas Pap cytology is a marker of additional risk in HPV-positive women, as shown in the Kaplan–Meier plots (Fig. 4). Our results are consistent with a long-term, retrospective analysis of 19,512 women attending a health maintenance programme, which showed that baseline Pap and HPV tests predicted CIN3+ risk within the first 2 years, but only HPV testing predicted long-term risk (10–18 years later).^21^

Although there was a notable difference between cohort sizes in round 1 and subsequent rounds, the data obtained per-protocol allow a reliable comparison over time. We identified 28 cases of cervical cancer in round 1, while another 3 cases were observed beyond round 1. The three cervical cancers detected in the second and third screening rounds could be explained by failure of screening tests or colposcopy, while the majority of CIN3 identified within round 2 and 3 were classified as new lesions in women who tested negative for HPV at recruitment. It is important to note that WOLPHSCREEN participants were regularly screened with cytology before recruitment to the pilot project^15^ and, therefore 25 of the 28 cancer cases detected during round 1 occurred in women whose previous screening had been done within the last 6 years and often annually.

A limitation of this analysis is loss to follow-up or indeterminate status of 2965 participants, mostly because of a change of insurance provider and subsequent difficulties in keeping track of young women among a highly mobile workforce; however, external clinical databases and registries were used to mitigate this issue and allowed us to identify additional diagnoses of cervical cancer made outside the protocol. Despite this loss to follow-up, data are available from almost 12,000 women with a mean follow-up of 1401 days or 3.84 years in subsequent screening rounds. To minimise the possibility that a similar reduction of cervical cancer incidence did not occur among participants who left or were excluded from WOLPHSCREEN, we searched all possible sources and identified just three cases of cervical cancer in that cohort.

An additional limitation of the analysis is that we could not directly compare CIN3 and cervical cancer data before and after the WOLPHSCREEN protocol was introduced. Although the reported incidence of cervical cancer in Wolfsburg could be used for such an analysis, CIN3 is not monitored reliably by German cancer registries making direct comparisons impossible. In addition, it is expected that a superior screening programme will lead to an increased incidence of cervical cancer and CIN3 in the first screening round because of better detection of cases, whereas the incidence will decrease in subsequent rounds, which is the effect that we observed in WOLPHSCREEN. As CIN3 is negatively associated with age, a decrease in incidence between the first and subsequent screening rounds may be anticipated. We did a sensitivity analysis, excluding women aged 30−34 years, which showed that the impact of ageing seems minor and the drop in CIN3 is not explained by 5 years' ageing of the population. Furthermore, the risk of invasive cancer does not have a linear association with age (peak incidence occurs among women aged 35–44 years) and we found that the decrease in cancer incidence (−78%) was almost as strong as the decrease in CIN3+ (−81%).

The analysis is robust and shows a reduction in the risk of invasive cervical cancer in women aged >30 years screened using HPV testing and risk-adapted follow-up. The introduction of HPV vaccination, which significantly reduces HPV-related lesions,^22^ is expected to decrease the risk of invasive cervical cancer and, in combination with HPV screening, may lead to eradication of cervical cancer.^23^ However, this effect is not likely to have occurred in our cohort already because vaccination started in 2007 in women aged <18 years and only exceptionally in older women. The COMPASS trial of cytology versus HPV testing in vaccinated women (aged ≤33 years) showed that HPV screening can increase detection of cervical precancerous lesions in vaccinated, as well as unvaccinated, populations.^24^ Nevertheless, despite the implementation of HPV vaccines, it is important to guard against complacency because cervical cancers are still occurring. Figure 3 reflects the challenges and limitations of HPV screening programmes. Although all observed cancers were associated with HPV and 28 of the 31 cancer patients tested HC2 positive at recruitment, only 14 cases were diagnosed <6 months after recruitment. Eleven of the 14 cancer patients diagnosed >6 months after primary screening in round 1 had normal Pap cytology initially and none of the other 3 cases had cytology findings indicating cancer (one ASC-H, two LSIL). For comparison, of the 14 cancers diagnosed within 6 months, only 2 had normal Pap, 12 had abnormal Pap, including 4 Pap IVa (HSIL/cells of CIN3) and 5 Pap V (cancer cells). These findings show that the major reason for late diagnoses was the delayed referral of women with HPV persistency but normal cytology for colposcopy, which underlines the importance of better triage concepts for this risk group. Furthermore, it is imperative that screening programmes are managed rigorously to avoid, or at least reduce, failures explained by deficient colposcopy. We already showed that the risk of CIN3+ missed by colposcopy is significantly increased in women with HPV persistency and normal cytology compared with women with abnormal cytology, especially in women with type 3 transformation zones.^13^ The results from this study suggest that an adjusted colposcopy protocol may allow a few hundred cancer cases per year to be avoided in Germany.

A recent systematic review by the US Preventive Services Task force^25^ found that primary high-risk HPV screening detected more CIN3+ at first screening compared with cytology while co-testing did not show initial increased CIN3+ detection in randomised controlled trials. We feel that the observed significant reduction of CIN3+ between the first and subsequent screening rounds in WOLPHSCREEN may be explained at least in part by our colposcopy protocol. Histological assessment was mandatory even in cases with minor changes and >93% of all women transferred for colposcopy received a histological diagnosis immediately, which may have avoided missing more CIN3 lesions at first colposcopy and changed the clinical course of some of the cases with HPV persistency. We observed that taking biopsies significantly increased the regression rate of persistent HPV infections compared with women who were followed without biopsies.^26^

While a negative HPV test can identify individuals not at high risk of developing invasive cervical cancer, a positive HPV test signals the need for appropriate intervention. Improving outcomes depends on the effectiveness of follow-up procedures and treatments to manage women identified with persistent HPV infection, cytological abnormalities and precursor lesions. The quality of colposcopy is particularly important because all screening analyses are based on the rate of lesions detected by colposcopy and not on the true rate of lesions. We showed previously that failure of colposcopy to detect prevalent CIN3 in women with abnormal cytology is significantly lower than in HPV-positive women with normal cytology.^17^ Overlooked CIN3 lesions or delayed diagnoses because of false-negative colposcopy in HPV-positive women with normal cytology will directly result in underestimation of the sensitivity of HPV-based screening programmes and miss the chance to increase the number of women at risk who are protected from cervical cancer. Therefore, it seems to be a major challenge for all new HPV-based screening programmes to identify and eliminate factors that may result in a delayed or missed diagnosis of CIN3+ lesions by colposcopy.

In conclusion, the observed decline in disease rates with 5-yearly co-testing seems mainly attributable to HPV testing since no CIN3+ occurred in HPV−/Pap+ women. These findings support routine HPV screening outside the setting of randomised controlled trials, although a formal assessment of benefits and harm is still required.

## Data Availability

WOLPHSCREEN is managed by Klinikum Wolfsburg, the central database is located in the department of gynaecology. The contract between health insurances, gynaecologists in private practice, other partners and Klinikum Wolfsburg encourages the use of WOLPHSCREEN data for research and gives all rights for analyses and publications to the head of the department of obstetrics and gynaecology as leader of the scientific team.
